# Supplementary material for: WordCommentsAnalyzer: A windows software tool for qualitative research
Source: F1000Res. 2018 Sep 4;7:536. Originally published 2018 May 3. [Version 2] doi: 10.12688/f1000research.14819.2 (PMC6137411; doi:10.12688/f1000research.14819.2)
Supplement: Supplementary file 3 [file f1000research-7-17614-s0002.tgz › b2a79496-4ada-47c4-9682-a99ce867836d.zip!/Eight Iranian Health Journals/Salmand_Iranian Journal of Ageing _21.docx]

© Salmand_Iranian Journal of Ageing . This work is licensed under CC BY-NC 4.0 (https://creativecommons.org/licenses/by-nc/4.0/).

Volume & Issue: دوره 8 - شماره 1

Title: وضعیت سلامت شناختی و سلامت عمومی در سالمندان با و بدون سابقه افتادن

http://salmandj.uswr.ac.ir/article-1-505-fa.html

اهداف: افتادن یک مشکل اصلی در سیستم های مراقبتی است و حدود %30 سالمندان، سالانه افتادن را یکبار یا بیشتر تجربه می‌کنند. عملکرد شناختی به‌عنوان یکی از ریسک فاکتورهای کلیدی در افتادن سالمندان محسوب می‌شود. در این مطالعه سعی بر آن داریم که وضعیت سلامت عمومی و شناختی سالمندان با و بدون سابقه افتادن را مقایسه کنیم. مواد و روش‌ها: در این مطالعه 22 سالمند بدون سابقه افتادن (15 مرد و 7 زن) با 31 سالمند با سابقه افتادن (21 مرد و 10 زن) توسط پرسش‌نامه سلامت عمومی و آزمون ام ام اس ای مورد ارزیابی و مقایسه قرار گرفتند. آزمون تی مستقل جهت آنالیز داده‌ها استفاده شد. یافته‌ها: یافته‌ها نشان داد که نمره کلی سلامت عمومی (0/009=P) و مقیاس فرعی اضطراب و اختلال خواب (0/004=P) بین دو گروه تفاوت معنی‌داری دارند. مقایسه میانگین‌ها نشان داد که سالمندان با سابقه افتادن اختلال بیشتری نسبت به سالمندان بدون سابقه افتادن از نظر نمره کلی و اضطراب و اختلال خواب داشتند. نمره کلی سلامت شناختی در دو گروه با و بدون سابقه زمین افتادن تفاوت معنی‌داری دارد (0/022=P)، هر چند که این تفاوت در هیچ یک از مقیاس‌های فرعی آزمون مشاهده نشد. مقایسه میانگین‌ها نشان داد که اختلال شناختی در سالمندان با سابقه افتادن نسبت به سالمندان بدون سابقه افتادن بیشتر است. نتیجه‌گیری: در این مطالعه ارتباط اضطراب و اختلال خواب با افتادن اثبات شد. اختلال در خواب می‌تواند با ایجاد حالت گیجی کلی منجر به افتادن شود. اضطراب و ترس از افتادن منجر به کاهش مشارکت افراد در فعالیت های فیزیکی معمول، تعادل، و افزایش خطر افتادن می شود که با روش‌های درمانی کاهش اضطراب می توان از افتادن پیشگیری کرد. به‌علاوه احتمالا درمان‌های شناختی در دوران سالمندی نیز منحر به کاهش خطرافتادن می‌شود.

Title: وضعیت سلامت معنوی در سالمندان شهر اصفهان

http://salmandj.uswr.ac.ir/article-1-364-fa.html

اهداف: در سالهای اخیر، دنیا بخصوص کشورهای در حال توسعه ای نظیر ایران افزایش جمعیت سالمندی را تجربه کرده است. لذا حفظ و ارتقای سلامت وکیفیت زندگی سالمندان به عنوان یک گروه جمعیتی آسیب‌پذیر بیش از پیش بایستی مورد توجه قرار گیرد. بدیهی است که شناسایی وضعیت سلامت سالمندان اولین گام جهت ارتقای سلامت این قشر می‌باشد. سلامت معنوی یکی از ابعاد مهم سلامت است که کمتر در گروه سالمند مورد توجه قرار گرفته است. هدف از انجام این مطالعه تعیین وضعیت سلامت معنوی سالمندان شهر اصفهان بوده است. مواد و روش‌ها: این مطالعه مقطعی بر روی 230 سالمند ٦٠ سال به بالا شهر اصفهان انجام شد. نمونه ها به روش در دسترس از همه  سطح شهر انتخاب شدند. داده‌ها توسط پرسشنامه ی مشتمل بر دو بخش ویژگی‌های دموگرافیک و ابزار سلامت معنوی Paloutzian و Ellison جمع‌آوری شد. جهت تجزیه و تحلیل اطلاعات از آزمون‌های تی مستقل، آنالیز واریانس، ضریب همبستگی و رگرسیون چندمتغیره استفاده شد. یافته‌ها: نتایج نشان داد 9/70 درصد ازافراد مورد مطالعه مرد بودند. میانگین سنی شرکت‌کنندگان مورد مطالعه 5/67 سال بوده است. میانگین نمره سلامت معنوی افراد مورد مطالعه 13/98±95/2 بود که مطلوب بوده است. سالمندانی که در خانه سالمندان سکونت داشتند از سطح سلامت معنوی پایین‌تری نسبت به بقیه گروه‌ها برخوردار بودند. بین میانگین نمره کل سلامت معنوی با متغیرهای سطح تحصیلات (0/05>P) و وضعیت سکونت (0/05>P) از نظر آماری ارتباط معنی‌دار وجود داشت. نتیجه‌گیری: حمایت ناشی از منابع معنوی یا مذهبی و داشتن ارتباط با قدرتی بالاتر سودمند بوده و می‌تواند برای بهبود کیفیت زندگی و ارتقای سلامت مفید باشد. با توجه به در نظر گرفتن سالمندان به عنوان قشر آسیب‌پذیرتر جامعه، نیاز به برنامه‌ریزی آموزشی و خدمات مشاوره‌ای در این زمینه احساس می‌گردد.

Title: ساخت و هنجاریابی مقیاس درجه‌بندی سنجش استرسورهای دوره سالمندی

http://salmandj.uswr.ac.ir/article-1-613-fa.html

هدف: هدف از انجام پژوهش حاضر ساخت و هنجاریابی مقیاس درجه‌بندی جهت شناسایی استروسورهای سالمندی است. مواد و روش‌ها: روش اجرا بدین صورت بود که چک لیستی شامل عوامل استرس زا در دوره سالمندی از چند منبع شامل مقیاس‌های درجه‌بندی سنجش استرس، چک لیست های مربوط به مسائل سالمندی، پرسش‌نامه‌ها، منابع مکتوب، مصاحبه با سالمندان و همچنین استفاده از نظر متخصصان شامل 100 آیتم جمع آوری و پس از مطالعه متخصصان به 45 آیتم تقلیل و پس از اجرای پایلوت به 40 آیتم رسید و سپس در قالب یک مقیاس درجه بندی و همچنین پاسخ بلی و خیر تنظیم شد. جامعه پژوهش حاضر کلیه سالمندان بالاتر از 60 سال هستند که در سال 1391 از خدمات بیمه استفاده می کنند. از این جامعه، 300 سالمند به روش هدفمند انتخاب و با کمک مقیاس مذکور مورد مصاحبه قرار گرفتند. پس از دو هفته 30 نفر از سالمندان مذکور مجددا با همین مقیاس، پرسش‌نامه GHQ و همچنین مقیاس سنجش هولمز و راهه مورد آزمون قرار گرفتند. یافته‌های پژوهش و تجزیه و تحلیل داده‌ها نشان می‌دهد. یافته‌ها: مقیاس نهایی دارای 6عامل است که مجموعاً 68/47درصد ازواریانس کل مقیاس را تبیین می‌کنند. محتوای مقیاس مورد تایید متخصصان و روایی سازه مقیاس با استفاده از ضریب همبستگی حاصل بین نمرات مقیاس و خرده مقیاس های GHQ تاییدکننده روایی مقیاس است. آلفای کرونباخ کل مقیاس برابر با 0/95 و ضریب پایایی به روش اسپیرمن- براون 0/84، به روش باز آزمون 0/79 و با استفاده از فرم مشابه (هولمز و راهه) برابر با 0/81 است. نقاط برش حاصل افراد را در 5 گروه دارای استرس بسیار کم، کم، متوسط، شدید و بسیار شدید تقسیم می کند، که توزیع افراد در هر طبقه منطبق با توزیع نظری نرمال می باشد. نتیجه‌گیری: در نتیجه مشخصات روان‌سنجی و همچنین نقاط برش حاصل استفاده همراه با اطمینان مقیاس را در موقعیت‌های پژوهشی توصیه می‌کند.

Title: بررسی وضعیت تغذیه سالمندان بستری در بیمارستان‌های آموزشی دانشگاه علوم پزشکی قزوین در سال 1390

http://salmandj.uswr.ac.ir/article-1-425-fa.html

اهداف: وضعیت تغذیه در سالمندی موضوع مهمی است که در کشورهای در حال توسعه به آن توجه کمی شده است. وضعیت تغذیه وابسته به ارتباط بین عوامل فردی و محیطی است و بر میزان مرگ، ناتوانی و کیفیت زندگی سالمندان تاثیر بسزایی دارد. لذا، این مطالعه با هدف تعیین وضعیت تغذیه سالمندان بستری در مراکز آموزشی- درمانی شهر قزوین انجام شد تا با شناخت عوامل مؤثر در این امر، اقدامات لازم برای ارتقاء سلامت سالمندان انجام گیرند. مواد و روش‌ها: این مطالعه مقطعی بر روی 233 سالمند (151 زن و 171 مرد) با سن بالاتر از 60 سال که در دو مرکز آموزشی- درمانی شهر قزوین بستری شده بودند، انجام شد. وضعیت تغذیه با استفاده از پرسش‌نامه MNA ارزیابی شد. افراد بر اساس نمره این پرسش‌نامه به گره‌های دچار سوء تغذیه، در معرض سوء تغذیه و بدون سوء تغذیه طبقه‌بندی شدند. یافته‌ها: در میان سالمندان مورد بررسی، %29/8 دارای وضعیت تغذیه مناسب بودند. %13/4 دچار سوء تغذیه و %42/95 در معرض سوءتغذیه بودند. تعداد افراد دچار سوءتغذیه در زنان بالاتر از مردان بود (%25/8 در مقابل %7/2، 0/001>P)؛ در حالی که میانگین شاخص توده بدنی در زنان بالاتر از مردان بود. وضعیت تغذیه با سن، شاخص توده بدنی، دور کمر و نسبت دور کمر به باسن ارتباط معنی‌داری داشت و عدم توانایی خروج از منزل، زندگی مستقل و مصرف روزانه مرغ و ماهی یا گوشت از عوامل خطر برای اختلال تغذیه شناخته شدند. نتیجه‌گیری: این مطالعه میزان بالای سوءتغذیه را در سالمندان بستری در بیمارستان نشان داد و ارزیابی وضعیت تغذیه در این افراد با استفاده از پرسش‌نامه MNA می توان به شناسایی این افراد و درمان جهت بهبود وضعیت آن‌ها کمک کند.

Title: مقایسه تعادل ایستا در مردان میانسال و سالمند فعال و غیر فعال

http://salmandj.uswr.ac.ir/article-1-122-fa.html

اهداف: هدف از مطالعه حاضر مقایسه تعادل ایستا در مردان میانسال و سالمند فعال و غیرفعال بود. مواد و روش‌ها: در این مطالعه مقطعی-مقایسه‌ای آزمودنی‌های تحقیق شامل دو گروه 55 مرد میانسال و سالمند فعال و 49 مرد میانسال و سالمند غیرفعال بودند. تعادل ایستا در آزمودنی‌ها با استفاده از آزمون ایستادن روی پا با چشمان باز و بسته اندازه‌گیری شد. سرعت (زمان) راه رفتن در آزمودنی‌های فعال با استفاده از آزمون پیاده‌روی راکپورت اندازه‌گیری شد. برای مقایسه تعادل ایستا با چشمان باز و بسته بین دو گروه از آزمون تی مستقل و برای بررسی ارتباط بین متغیرها از آزمون ضریب همبستگی پیرسون استفاده شد. تجزیه تحلیل داده ها با استفاده از نرم افزار SPSS16 انجام شد. یافته‌ها: نتایج نشان داد که تعادل ایستا با چشمان بسته و باز در گروه فعال به‌طور معنادری نسبت به گروه غیرفعال بالاتر می‌باشد. با این حال در گروه فعال هیچ‌گونه ارتباط معناداری بین زمان راه رفتن و میزان تعادل با چشم باز و بسته مشاهده نشد. یک همبستگی منفی و معناداری بین سن و تعادل ایستا مشاهده شد به‌طوریکه با افزایش سن تعادل به‌طور معنی‌داری کاهش می‌یافت. نتیجه‌گیری: یافته‌های مطالعه حاضر نشان داد که مردان میانسال و سالمند فعالی که به‌طور منظم به پیاده‌روی و ورزش صبحگاهی می‌پردازند نسبت به مردان میانسال و سالمند غیرفعال از تعادل بهتری برخوردار می‌باشند که این می‌تواند موید نقش تمرین به‌ویژه پیاده‌روی در بهبود تعادل باشد. احتمال می رود که پیاده‌روی با اعمال اضافه بار بر روی انتقال اطلاعات از طریق بهبود سیستم‌های حسی سه‌گانه دستگاه عصبی مرکزی و همچنین سیستم حرکتی جهت حفظ تعادل، باعث بهبود تعادل شود.

Title: بررسی حوادث در سالمندان استان کرمان طی سال‌های 88-1385

http://salmandj.uswr.ac.ir/article-1-424-fa.html

اهداف: سالمندان نیز نظیر سایر گروه‌های سنی در حوادث دچار آسیب و مرگ و میر می‌شوند منتهی علی رغم شرایط خاص جسمی و تغییرات دوره سالمندی متاسفانه در برخورد با حوادث، این گروه سنی کمترمورد عنایت قرار می‌گیرند. هدف از این مطالعه انعکاس وضعیت سالمندان استان کرمان در رابطه با حوادث می‌باشد. مواد و روش‌ها: مطالعه حاضر از نوع مقطعی می باشد و طی آن از میان تعداد 282618 مورد حادثه ثبت شده در طی سال‌های 1385 تا 1388 در استان کرمان (به استثنای شهرستان رفسنجان)، تعداد 11120 فرد 60 سال و مسن‌تر شناسایی شده و مورد بررسی قرار گرفتند. جهت تحلیل داده‌ها از آزمون‌های t و کای دو استفاده گردید. یافته‌ها: از کل افراد بررسی شده %63 مرد و %37 زن می‌باشند، میانگین سنی ایشان 7/97±69/05 بوده، بیشترین نوع حادثه مربوط به حوادث ترافیکی (%25/3) و پس از آن ضربه (%18/6) و سقوط (%11/1) بود. بیشترین موارد حادثه در منزل رخ داده (%41/7) و کوچه و خیابان نیز در رتبه دوم قرار دارند (%30/2). از کل آسیب‌دیدگان، 91 مورد مرگ (%0/82) رخ داده و مابقی زنده بوده و تحت درمان قرار گرفتند. تحلیل داده‌ها حاکی از آن است که خانم‌های آسیب دیده به‌طور معنی‌داری مسن‌تر از آقایان هستند (0/01>P). همچنین افراد فوت شده نیز از سن بالاتری برخوردار بودند (0/01>P). در حالیکه در مردان سالمند حوادث ترافیکی و خشونت بیشتر موجب آسیب شده، در خانم‌ها حوادثی نظیر سقوط، مسمومیت، عقرب و مارگزیدگی بیشتر رخ داده است (0/001>P). ازنظر محل وقوع حادثه نیز در مردان بیشتر حوادث در معابر ترافیکی و محل کار و اماکن عمومی رخ داده اند منتهی در خانم ها حوادث بیشتر در منزل به وقوع پیوسته اند(0/001>P). نتیجه‌گیری: حوادث در سالمندان به دلیل تغییرات ناشی از بالا رفتن سن، بیماری‌های همراه و مصرف داروها دارای اهمیت ویژه‌ای می‌باشند. آموزش در رابطه با پیشگیری و ایمن نمودن محیط منزل و جامعه می‌تواند به کاهش آسیب‌های مرتبط با حوادث کمک نموده و باید مدنظر قرار گیرند.

Title: بررسی تأثیر یک دوره تمرین استقامتی در آب بر سلامت عمومی روان مردان سالمند

http://salmandj.uswr.ac.ir/article-1-673-fa.html

اهداف: تمرین در آب یکی از مهم‌ترین فعالیت‌های توصیه شده به سالمندان محسوب می‌شود. هدف از پژوهش حاضر بررسی اثر یک دوره تمرین استقامتی در آب بر میزان سلامت روان، ابعاد نشانه‌های بدنی، اضظراب و بیخوابی، کارکرد اجتماعی و افسردگی مردان سالمند بود. مواد و روش‌ها: در این مطالعه نیمه تجربی 30 مرد سالمند به روش در دسترس انتخاب و به‌طور تصادفی در دو گروه کنترل و تجربی تقسیم شدند. ابتدا تمامی شرکت‌کنندگان پرسش‌نامه سلامت عمومی روان گلدبرگ (GHQ-28) را تکمیل نمودند. سپس گروه تجربی به مدت 6 هفته (سه جلسه در هفته) تمرین استقامتی در آب را اجرا کرده و گروه کنترل فعالیت معمول روزانه خود را اجرا نمودند. بعد از مداخله تمرینی هر دو گروه مجددا پرسش‌نامه را تکمیل نمودند. تحلیل داده‌ها با استفاده از تی مستقل و همبسته اجرا گردید. یافته‌ها: نتایج آزمون تی همبسته نشان داد که تمرین استقامتی در آب تاثیر معنی‌داری بر سلامت عمومی روان (0/000=P) و خرده مقیاس‌های نشانه‌های بدنی (0/000=P)، اضطراب و بی‌خوابی (0/034=P)، افسردگی (0/000=P) و کارکرد اجتماعی (0/000=P) در گروه تجربی دارد لیکن در گروه کنترل که تمرین استقامتی در آب را اجرا نکرده بودند تغییرات معنی‌دار نبود. همچنین نتایج آزمون تی مستقل نشان داد که تفاوت معنی‌داری (0/001=P) بین میانگین نمره کلی سلامت روان گروه کنترل و تجربی پس از مداخله تمرینی وجود دارد. نتیجه‌گیری: با توجه به یافته‌های این تحقیق می‌توان نتیجه گرفت که تمرین استقامتی در آب در ارزیابی روان‌شناختی سالمندان نسبت به سلامت عمومی روان، اضطراب و بی‌خوابی، نشانه‌های بدنی، کارکرد اجتماعی و افسردگی نقش مثبت و اثرگذاری دارد.

Title: بررسی میزان شیوع و عوامل مرتبط با زمین‌خوردن در سالمندان بالای 55 سال شهر شیراز

http://salmandj.uswr.ac.ir/article-1-548-fa.html

اهداف: یکی از شایع‌ترین مشکلات سالمندان، زمین‌خوردن است، زمین خوردن ممکن است بر بهداشت و سلامت جسمی و روحی فرد اثر بگذارد و برای جامعه و فرد یکسری مشکلات اقتصادی و اجتماعی ایجاد کند. در این مطالعه شیوع زمین‌خوردن در افراد کهنسال و عوامل مرتبط با آن بررسی شد.  مواد و روش‌ها: در یک تحقیق از نوع توصیفی-تحلیلی و به روش مقطعی، 750 فرد کهنسال بالای 55 سال ساکن شهر شیراز (409 زن، 34 مرد، میانگین سنی 67/32 سال) با روش نمونه گیری آسان، در مطالعه شرکت کردند. براساس اطلاعات مورد نیاز مطالعه، یک فرم جمع‌آوری اطلاعات تهیه شد که شامل اطلاعاتی در مورد مشخصات کلی فرد و سابقه زمین‌خوردن در یک سال گذشته و عوامل احتمالی مرتبط با زمین خوردن بود. آنالیز داده‌های آماری توسط آزمون‌های C‏hi-square ،Mann-Whitney و همچنین روش رگرسیون لجستیک انجام شد. یافته‌ها: میزان شیوع زمین خوردن در این مطالعه %33/6 برای افراد بالای 55 سال بود. %20/4 افراد فقط یک بار زمین‌خوردن داشتند و %13/2 زمین خوردن مکرر داشتند بین زمین‌خوردن افراد با سن (0/016 =P)، جنسیت (0/001>P)، قند خون بالا (0/008=P) و سرگیجه (0/001>P) از لحاظ آماری ارتباط معنی‌داری وجود داشت. اما بین زمین خوردن افراد با بیماری سیستم عصبی، بیماری روماتیسمی، فشار خون بالا، آرتروز، بیماری قلبی، استفاده از عصا و استفاده از عینک از لحاظ آماری ارتباط معنی‌داری وجود نداشت. (0/05=P) و پوکی استخوان (0/024=P) وجود داشت. همچنین استفاده از عصا (0/023=P) و آرتروز (0/048=P) با زمین خوردن همراه با آسیب ارتباط داشتند.  نتیجه‌گیری: نتایج حاصل از مطالعه فوق بیانگر این مطلب است که یک سوم افراد بالای 55 سال مقیم جامعه در طی یک سال زمین افتادن را تجربه می کنند و عواملی از جمله قند خون بالا و سرگیجه خطر زمین خوردن را در سالمندان به‌طور چشمگیری افزایش می‌دهند. با برنامه‌ریزی جهت کنترل این عوامل می‌توان میزان زمین‌خوردن کهنسالان و عوارض و مشکلات بعد از آن را کاهش داد.
